# A rare case of eczema herpeticum associated with HIV: A case report

**DOI:** 10.1016/j.idcr.2022.e01660

**Published:** 2022-12-05

**Authors:** Prabal Chourasia, Norman D. Bernstein, Kunal M. Ajmera, Trupti Pandit, Ramesh Pandit, Lokesh Goyal

**Affiliations:** a Mary Washington Hospital, Fredericksburg, VA, USA; b Sentara Northern Virginia Medical Center, Woodbridge, VA, USA; c Christiana Care, Delaware, USA; d University of Pennsylvania, USA; e Christus Spohn Hospital shoreline, Corpus Cristi, Texas, USA

**Keywords:** Eczema herpeticum, HIV, Steroid use, Kaposi’s varicelliform eruption, Acyclovir

## Abstract

Eczema herpeticum (EH), also known as Kaposi's varicelliform eruption, is a disseminated herpes simplex virus infection seen in patients with underlying skin conditions, most commonly atopic dermatitis. Monomorphic vesicles and "punched-out" erosions with hemorrhagic crusts over eczematous regions are the hallmarks of EH's presentation. Here, we discuss a first reported case of eczema herpeticum in a patient living with well controlled HIV with prior steroid use. A 30-year-old male patient living with HIV presented to the hospital with a generalized rash involving the face, neck, arms, hands, low back region, and both feet. Herpes simplex 1 and 2 by PCR DNA were detected from external auditory ear canal drainage. The patient was treated with intravenous acyclovir and responded well. He had long term history of eczema and required acyclovir prophylaxis later.

## Introduction

Eczema Herpeticum (EH) or Kaposi's varicelliform eruption is a rare but severe form of herpes simplex infection involving damaged skin sites [1], [2]. It is seen most commonly in atopic dermatitis though its association with other immunological diseases like cutaneous T cell Lymphoma [3], and Sezary disease has been reported [4]. The differential diagnosis includes impetigo, primary varicella infection, herpes zoster, smallpox, irritant or allergic contact dermatitis and autoimmune blistering disease. EH is a skin eruption that may manifest as an acute, generalized vesiculopustular rash that must be distinguished from smallpox and, more recently, monkeypox [5]. The Centers for Disease Control (CDC) has created clinical algorithms for the rapid evaluation of individuals with acute widespread vesiculopustular rash illness (AGV-PRI) [6].

Cutaneous herpes virus infection in immunocompetent patients may resolve in a few days. On the other hand, immunocompromised individuals, such as those living with HIV and cancer, can experience severe morbidity and mortality (up to 50%)[7]. Superimposed bacterial and fungal infections can further complicate this condition [8]. The prompt recognition of EH in immunosuppressed patients, especially with history of prolonged steroid use, is critical to lower viral dissemination, superinfection, and associated morbidity and mortality [9], [10]. Association of EH in patients living with well controlled HIV has been rarely reported. Another case of EH was reported in an woman living with HIV after skin resurfacing with a laser [11]. To our knowledge, this is the first description of EH in a patient living with well controlled HIV disease with prior steroid use. It should be on the differential diagnosis of vesiculopustular rash in patients living with HIV and prior steroid use.

## Case report

**Fig. 1:**
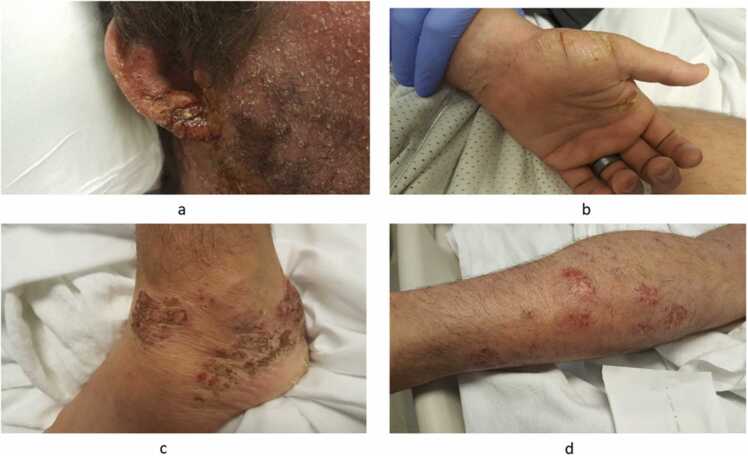
Diffuse Erythematous rash showing involvement in a. Earlobe b. Hand c. Leg d. Ankle.

A 30-year-old male with a long-standing history of living with HIV on appropriate antiretroviral therapy presented to the hospital emergency department for evaluation of worsening generalized itchy and painful erythematous rash involving entire face (including around ears), neck, arms, hands, feet and trunk that had developed over a month (Fig. 1). He also complained of soreness in the throat with right ear drainage and purulent eruptions. His past medical history included a skin biopsy for his recalcitrant eczema which showed spongiotic dermatitis. This rash was extensively evaluated in the past with negative PAS stain for fungus, immunofluorescence study, ANA test, anti-mitochondrial antibody, ANCA, celiac antibody test, stool for Entamoeba histolytica/other enteric pathogens (including cryptosporidium and giardiasis), strongyloides antibody serology, urinary Chlamydia and Neisseria gonorrhea testing. The patient had a history of positive CMV antibodies, eosinophilia and elevated IgE levels. The patient was treated with steroids for a prolonged time, eventually stopped due to concern for long-term side effects.

The patient had a febrile course. Physical examination was notable for diffuse areas of erythema with monomorphic confluent erosions with yellow crusting, dryness and cracking on the face, neck, palms of the hands, trunk, lower back region and left foot. The patient did not have any lymphadenopathy, conjunctivitis, or neck rigidity. Patient’s laboratory workup is presented in the Table 1.

**Table 1:** Patients laboratory work up during the hospital stay.

Day	Lab	Reference range	Value
Day 1	WBC	4.50–11.00 K/UL	5.47
Day 2	Eosinophil count	50–400 K/UL	610 (increased)
	IgE total	< 214 kU/L	9750 (increased)
	Zinc	0.66–1.10(mcg/ml)	0.60(decreased)
	Vitamin B12	239–931 pg/ml	184 (decreased)
	Wound culture from facial vesicles	N/A	Enterobacter cloacae resistant to multiple antibiotics including Augmentin, ceftazidime and Zosyn
Day 9	HSV PCR from right ear swab		Positive for HSV-1 and HSV-2 target DNA
	CD4 cell count	365–1437 cells/mcL	370
	CD8 cell count	171–846 cells/mcL	213
	CD45 lymphocyte count	0.82–2.84 thou/mcl	0.64
	CD3 (T cells)	58–86%	92%
	HIV-RNA by PCR,Qn		< 20 copies/ml
Day 10	HSV1 IgG		Negative
	HSV2 IgG		Positive
Day 16	HSV IgM, EIA		Reactive
	HSV IgM, EIA		

WBC-white blood cells, HSV-Herpes Simplex Virus, PCR-polymerase chain reaction.

The patient had normal liver and kidney function tests, iron panel, folic acid, and B1/B6 levels. The patient's blood culture did not show any growth. The patient had negative nasal MRSA screen, perirectal VRE, QuantiFERON TB, RPR, Strongyloides antibody IgG, syphilis IgG antibody, hepatitis B surface antigen/antibody, hepatitis B core total antibody, hepatitis C antibody test, hepatitis C quantitative RNA. CT of face and neck on day 5 of the presentation was notable for prominent lymph nodes, most located at left of the midline in the sublingual region, pathological enlarged submental lymph node to left, and borderline enlarged right-sided level 2 cervical lymph node throughout cervical chains.

The patient was admitted with a provisional diagnosis of possible atopic dermatitis with cellulitis of face and neck and started on weight-based intravenous vancomycin but left against medical advice. He was readmitted the next day with ongoing symptoms. He declined another skin biopsy as the previous biopsy did not clarify an entity beyond his original eczema. A dermatologist at a different facility assessed his images and suggested collecting Herpes viral PCR samples from any drainage locations. Given the findings, the patient was placed on intravenous acyclovir due to the high likelihood of EH. His drainage from the right external auditory ear canal was positive for both HSV 1 and 2 by PCR DNA. Intravenous antibiotic coverage for superinfection was also continued until the patient course stabilized. The patient's symptoms and rash improved significantly after being started on intravenous acyclovir. The patient was eventually transitioned to oral valacyclovir and clindamycin at discharge time. Patient was maintained on oral valacyclovir for prophylaxis. For the past 2 years, a consultant dermatologist has prescribed dupilumab with success to treat his eczema. Most recently, the patient has been placed back on valacyclovir treatment and then prophylaxis because of relapsing herpes infection. The patient has been followed up for 6 years.

## Discussion

EH is a viral skin infection most commonly caused by HSV-1 or HSV-2 that complicates a preexistent skin disorder and may lead to both skin and visceral dissemination if left undiagnosed and untreated [12]. It is considered a dermatological emergency and requires a high level of suspicion for diagnosis [9], [13]. Early diagnosis can lead to effective treatment, whereas delay in diagnosis can lead to complications, including viremia, bacteremia and death [13]. It is mostly associated with atopic dermatitis but has been described in other skin conditions like Darier disease [14], pemphigus foliaceus [15], mycosis fungoides, Sezary syndrome [4], ichthyosis vulgaris [16],Hailey-Hailey disease [17] and severe combined immunodeficiency[18]. While EH’s association with multiple cutaneous conditions has been reported before, its association in patients with well controlled HIV with steroid use has rarely been described. To the best of our knowledge, this is the first description of EH in people living with well controlled HIV disease with prior steroid use.

Pathophysiology of EH includes impairment of skin barrier, decreased production of antimicrobial peptides cathelicidin and human β-defensins, overexpression of cytokines, downregulation of type 1–3 interferons, and defect in immune cell function [1]. Other factors which can contribute to the development of EH include genetic polymorphisms, elevated total IgE level, AD topical calcineurin inhibitor treatment, and staphylococcal colonization with toxin secretion promoting HSV replication [1], [12]). In our patient, HIV and prior steroid use may have contributed to decreased immunity and increased susceptibility to herpes virus.

EH is a clinical diagnosis with patients presenting with disseminated monomorphic dome shaped vesicles along with malaise and lymphadenopathy [12]. These vesicles may develop into pustules followed by crusts filling erosive pits. The most affected body parts are the head, neck, and upper parts of the body. Lesions may heal in 2–6 weeks. Other symptoms and signs that can be seen in EH include fever and lymphopenia. With characteristic rash morphology and clinical signs, the diagnosis of EH can be confirmed by positive polymerase chain reaction of HSV viral DNA of vesicle fluid without skin biopsy. This laboratory method for identification of the presence of virus is a gold standard with high sensitivity and specificity [19]. Other tests that can be considered if PCR is not available are Tzank smear, electron microscopy or commercial immunofluorescence tests [12], [19]. Skin biopsy can be considered for atypical cases. Differential diagnosis includes impetigo, contact dermatitis, chicken pox, now Monkeypox and Stevens-Johnson syndrome, among other skin conditions with cutaneous dissemination [12]. Elevated ESR is more common in patients who are colonized with secondary bacterial infection [12]. Bacterial culture should be performed if there is concern for superinfection [19]. In our patient, diagnosis was suspected clinically and confirmed by drainage from ear by PCR.

Early diagnosis and treatment are associated with good outcomes. [10], [13], [20] Acyclovir has been a mainstay of treatment[10], [20]. Initial systemic acyclovir therapy and hospitalization are recommended for immunocompromised patients and those with severe disease [1], [7], [21], [20], [22]. Our patient did not initially respond to intravenous antibiotics. He responded well to intravenous acyclovir. Given EH is frequently associated with secondary infection most commonly staphylococcus, antibiotics are often initiated as part of initial treatment [22].

## Conclusion

EH has rarely been described in patients living with well controlled HIV and prior steroid use. EH is more prevalent in those with severe atopic dermatitis. In immunocompromised people, it can cause a life-threatening infection. Reducing EH-related morbidity and mortality requires prompt diagnosis and treatment with acyclovir. Bacterial superinfection may be a complication of the clinical course. This condition should be suspected in all immunocompromised patients including in patients living with well controlled HIV and steroid use who present with diffuse rash associated with fever and lymphadenopathy. Early antiviral treatment with intravenous acyclovir is key to reducing morbidity and mortality associated with EH.

## Funding support

This research did not receive any specific grant from funding agencies in the public, commercial, or not-for-profit sectors.

## CRediT authorship contribution statement

PKC and NB: data collection, manuscript preparation, NB: Involved in patient care, LG, TP, KA and RP: manuscript preparation and review.

## Ethical approval

Not applicable.

## Consent

Authors have patients consent to use case information and images.

## Declaration of interest

None.
